# Supplementary material for: Rheological Properties, Particle Size Distribution and Physical Stability of Novel Refined Pumpkin Seed Oil Creams with Oleogel and Lucuma Powder
Source: Foods. 2022 Jun 22;11(13):1844. doi: 10.3390/foods11131844 (PMC9266185; doi:10.3390/foods11131844)
Supplement: Supplementary file 1 [file foods-11-01844-s001.zip › foods-1779297-supplementary.pdf]

Supplementary data

FIGURE S1

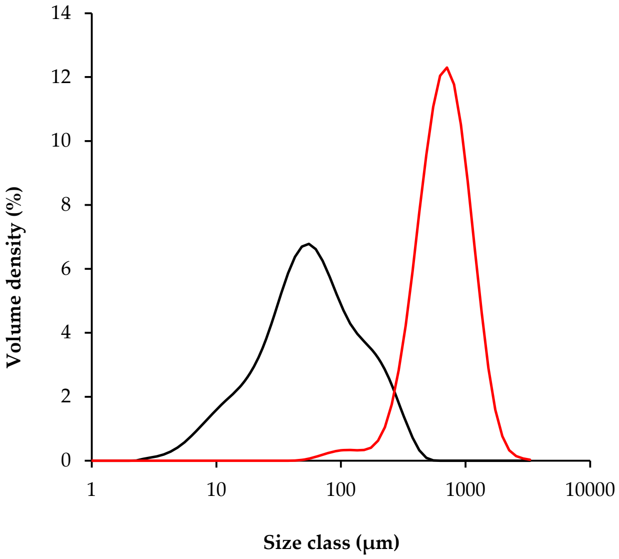

Figure S1 Particle size distribution (PSD) of saccharose ( )and Lucuma powder ( ).

TABLE S1

Table S1 Mean values (±standard error) of D10, D50, D90 for creams CBS, OS and OLS at different refining times.

Cream	Refining time (min)	D10 (µm)	D50 (µm)	D90 (µm)
CBS	60	2.60±0.01	10.86±0.14[d]	86.49±5.76[d]
	90	2.49±0.04	9.89±0.11[c]	51.56±1.00[c]
	120	2.49±0.03	9.08±0.13[b]	35.66±1.40[b]
	150	2.42±0.00	8.27±0.12[a]	26.41±1.43[a]
OS	60	2.71±0.01	11.62±0.04[d]	66.64±0.43[c]
	90	2.64±0.01	10.12±0.05[c]	42.37±0.34[b]
	120	2.55±0.00	9.04±0.01[b]	31.37±0.25[a]
	150	2.21±0.06	7.76±0.11[a]	24.38±0.22[a]
OLS	60	2.97±0.14	16.02±2.20[d]	87.63±0.98[d]
	90	2.61±0.31	12.60±0.24[c]	56.67±0.50[c]
	120	2.72±0.03	11.38±0.08[b]	46.74±0.25[b]
	150	2.55±0.00	10.14±0.00[a]	37.39±0.07[a]

Different letters correspond to significantly different values (p<0.05) from Duncan's test. The letters are missing where no significant differences between the samples were observed.
